# Serum β2-microglobulin as a predictor of residual kidney function in peritoneal dialysis patients

**DOI:** 10.1007/s40620-020-00906-x

**Published:** 2020-12-03

**Authors:** David A. Jaques, Andrew Davenport

**Affiliations:** 1 Division of Nephrology, Geneva University Hospitals, Rue Gabrielle-Perret-Gentil 4, 1205 Geneva, Switzerland; 2 UCL Department of Nephrology, Royal Free Hospital, University College London, London, UK

**Keywords:** Peritoneal dialysis, Residual renal function, Serum β2-microglobulin, Incremental dialysis

## Abstract

**Background:**

While clinical guidelines recommend that residual kidney function (RKF) is measured in peritoneal dialysis (PD) patients, 24-h urine collection is cumbersome and prone to errors. We wished to determine whether an equation using serum β2-microglobulin (β2M) could prove of clinical benefit in estimating RKF and identifying patients who could start PD with incremental prescriptions.

**Methods:**

We measured serum β2M in consecutive PD outpatients recently starting dialysis with continuous ambulatory PD (CAPD) or automated PD (APD), attending a single tertiary hospital for their routine clinical visit. RKF was defined as the mean of 24-h urine clearances of creatinine and urea. An equation estimating RKF (eRKF) was generated based on serum β2M levels on a randomly selected modelling group.

**Results:**

We included 511 patients, of whom 351 in the modelling group and 150 in the validation group. Mean age was 58.7 ± 15.8, 307 (60.0%) were men and median RKF value was 4.5 (2.4–6.5) mL/min/1.73 m^2^. In the validation group, an equation based on β2M, creatinine, urea, age and gender showed minimal bias of − 0.1 mL/min/1.73 m^2^ to estimate RKF. Area under the receiving operator characteristic curve was 0.915 to detect RKF ≥ 2 mL/min/1.73 m^2^.

**Conclusion:**

An equation based on serum β2M concentration would not be able to replace 24-h urine collection as the standard of care when an exact measurement of RKF is required. However, it could prove useful in identifying patients suitable for an incremental PD prescription and for monitoring RKF in individuals unable to reliably collect urine.

**Graphic abstract:**

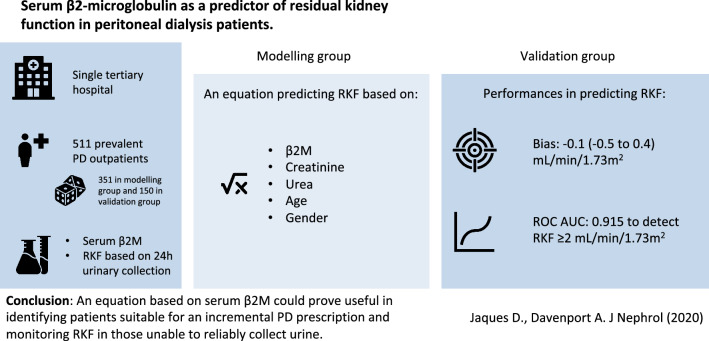

## Introduction

Residual kidney function (RKF) is a major prognostic factor for peritoneal dialysis (PD) patients, associated with mortality, morbidity and quality of life [1, 2]. Patients with preserved RKF have been reported to have better maintenance of euvolemia, blood pressure (BP) control, improved nutritional status, reduced erythropoietin requirements, less systemic inflammation and lower risk of peritonitis [3, 4]. Consequently, most recent clinical guidelines recommend that RKF should be determined regularly in all PD patients [5–7]. In addition, if patients have significant RKF at the initiation of PD, this may allow them to have a less intense incremental prescription that could be increased later as RKF is lost [5–7]. However, not all patients are capable of collecting urine and even so, 24-h urine collections are cumbersome and potentially unreliable, so alternative methods using endogenous serum markers to estimate RKF are desirable [8].

Serum concentrations of low-molecular weight proteins (LMWP), including Cystatin C, β-trace protein and β2-microglobulin (β2M) are of interest in this setting as they are efficiently removed by renal clearance but less so by PD clearance [9–11]. A limited number of studies have assessed the ability of equations to predict RKF in PD patients, with those using LMWPs generally reporting better performance compared to those using small solutes [12–15]. However, removal of LMWPs differ according to dialysis modality and previous studies in PD patients tended to focus exclusively on continuous ambulatory PD (CAPD) or combined haemodialysis (HD) and PD patients [12, 13, 15–19].

Based on the current state of knowledge, the clinical utility of LMWP-based equations to estimate RKF and guide the management of real-world PD patients is unclear. As such, we wished to conduct a retrospective study using β2M as the most widely available LMWP in order to (i) describe the clinical determinants of β2M concentration in PD patients and (ii) assess the clinical applicability of a β2M-based equation to predict RKF and identify patients recently starting PD who could benefit from an incremental approach with a less intense treatment.

## Methods

### Selection of participants

We consecutively included PD outpatients who recently started dialysis attending a single tertiary hospital for their routine clinical follow-up and assessment of peritoneal membrane function. PD modalities were CAPD and automated PD (APD). APD comprised nocturnal intermittent PD (NIPD), continuous cycling PD (CCPD) and continuous optimized PD (COPD). Exclusion criteria were (i) RKF ≥ 15 mL/min/1.73 m^2^ as these patients were not considered relevant to the clinical question of the study, (ii) peritonitis or admission to hospital in the previous 12 weeks, (iii) limb amputation, stroke or other neuromuscular disease leading to limb atrophy, (iv) chronic liver disease with clinical ascites, (v) pregnancy and (vi) chemotherapy for cancer. No patient was prescribed a glucose dialysate concentration above 2.27%.

### Collection of variables

Peritoneal membrane characteristics were evaluated using the peritoneal equilibrium test (PET) from plasma creatinine concentration and a 4-h dwell with 2 L of 2.27% dialysate [20]. Transport type was defined according to European guidelines as slow, average and fast [21]. PD adequacy was calculated by standard methods from 24-h urine collections and samples from all spent dialysates [22]. Multifrequency bioelectrical impedance (MFBIA) was measured using a standardised protocol (InBody 720, Seoul, South Korea), with dialysate drained out and after voiding to determine body composition [23]. Skeletal muscle mass (SMM) was indexed (SMMI) and defined as SMM divided by the square of body height (kg/m^2^).

### Measurement of creatinine, urea and β2M

Creatinine and urea were measured by standard biochemical and enzymatic methodology (Roche Modular P, Roche Diagnostics, Lewes, UK). Serum β2M was measured by an immunoturbidimetric assay (Roche Cobas c702, Roche Diagnostics, Lewes, UK). All laboratories were UK accredited, and creatinine measurements were aligned by isotope dilution mass spectrometry (IDMS) standards (IDMS).

### Measurement of RKF

We used the following equation to measure creatinine and urea clearances based on a 24-h urine collection:

$$Clearance\;{\text{(mL/min)}} = \frac{Uvol \times Ucon}{{Pcon \times T}}$$

where *Uvol* is urine volume, *Ucon* is urine concentration, *Pcon* is plasma concentration and T is collection duration. According to the most recent guidelines, RKF was measured as the mean of creatinine and urea clearances [5–7]. RKF was then normalized to body surface area (BSA) using the Haycock formula and expressed as RKF (mL/min/1.73 m^2^).

### Statistical analysis

Continuous variables are expressed as mean and standard deviation (SD) or median and interquartile range (IQR) according to distribution while categorical variables are expressed as number and relative frequencies. Normality of distribution was assessed graphically. No outliers were specified. Variables were compared between groups using Student’s *t*-test and Chi-square for continuous and categorical variables, respectively. For regression models, linearity of relationship, normality of residuals and homoscedasticity of residuals were assessed graphically. Results are presented as β coefficients and associated 95% confidence intervals (95% CI) as well as *p*-values. A two-sided *p*-value < 0.05 was considered significant. Statistical analyses were conducted using STATA version 15 (StataCorp, 4905 Lakeway Drive, College Station, Texas 77845 USA).

### Determination of predictors of β2M

A multivariate linear regression model was used with β2M as the dependent variable and the following a priori selected independent variables: age, gender, ethnicity (Caucasian vs non-Caucasian), smoking status, body surface area (BMI), diabetes, C-reactive protein (CRP), use of furosemide, Davies comorbidity score, RKF, months of PD treatment (dialysis vintage), PD Kt/V urea, normalised protein nitrogen appearance (nPNA), 24-h ultrafiltration (UF), use of icodextrin, use of 2.27% dialysate, PD mode (CAPD vs APD), transport type (slow, average and fast), dwell volume and number of cycles. A backward stepwise process was then applied sequentially keeping only independent variables with *p*-values < 0.05 in the model.

### Construction of predictive equation for RKF

Patients were randomly divided into two groups: A modelling group used to construct a predictive equation estimating RKF (eRKF) and a validation group used to assess equation performances to predict measured RFK. Equations were generated using the multivariable fractional polynomial (MFP) method [24]. Briefly, MFP allows backwward elimination of possible predictors and selection of a fractional polynomial (FP) function accounting for the non-linear relationship of continuous variables. To avoid overfitting, allowed powers were − 2, − 1, − 0.5, 0 (corresponding to log transform by definition), 0.5, 1 and 2, while maximum degree of FP was 2. To obtain a reasonably parsimonious model, *p*-values for inclusion of covariates and determination of significance of FP transformation were 0.2 and 0.05, respectively. The following potential predictors were a priori considered: β2M, creatinine, urea, age, gender, ethnicity and CRP.

### Assessment of predictive equation for RKF

The number of patients in the validation group was pre-specified at 150. Correlation between RKF and eRKF was assessed with Spearman’s and Pearson’s correlation coefficients. Level of agreement between RKF and eRKF at various cut-off values was assessed using the kappa statistic and Bland and Altman analysis. Bias was defined as the median of the difference between RKF and eRFK. Precision was defined as the IQR of the difference between RFK and eRKF. Accuracy was defined as the percentage of eRKF estimates within ± 2 mL/min/1.73 m^2^ of RKF. Finally, receiving operator characteristic (ROC) analysis was performed for prediction of various cut-off values of RKF.

### Ethics

Our retrospective audit was checked with, and complied with the United Kingdom (UK) National Health Service Health Research Authority guidelines for clinical audit and service development (https://www.hra.nhs.uk), and registered with the UCL Department of Nephrology Royal Free Hospital. All patient data were anonymised.

## Results

The study cohort consisted of 511 patients. 361 patients were randomly attributed to the modelling group and 150 to the validation group. Mean age was 58.7 ± 15.8, and 307 (60.0%) were men. Median dialysis vintage was 2 (2–3) months. Mean β2M and median RKF values were 23.8 ± 10.9 mg/L and 4.5 (2.4–6.5) mL/min/1.73 m^2^, respectively. Patient’s characteristics according to random grouping are described in Table 1. Compared to the modelling group, patients in the validation group were more frequently slow transporters and had higher dwell volumes.

**Table 1:** Patient’s characteristics

Characteristics	Overall (*N* = 511)	Modelling group (*N* = 361)	Validation group (*N* = 150)	*p* value
Clinical characteristics				
Age (years)	58.7 ± 15.8	58.7 ± 16.0	58.7 ± 15.5	0.965
Gender (men)	307 (60.0%)	212 (58.7%)	95 (63.3%)	0.333
Ethnicity (Caucasian)	222 (43.4%)	158 (43.7%)	64 (42.6%)	0.819
BMI (kg/m^2^)	26.4 ± 5.3	26.6 ± 5.4	25.8 ± 4.9	0.130
Diabetes	223 (43.6%)	161 (44.6%)	62 (41.3%)	0.498
Hypertension	413 (80.8%)	287 (79.5%)	126 (84.0%)	0.239
Smoking	71 (13.9%)	52 (14.4%)	19 (12.6%)	0.589
Davies Score	1.2 ± 1.0	1.2 ± 1.0	1.1 ± 1.0	0.868
SBP (mmHg)	141.7 ± 24.3	142.1 ± 23.5	140.7 ± 26.2	0.544
DBP (mmHg)	82.2 ± 14.9	82.4 ± 15.3	81.6 ± 13.9	0.564
Laboratory characteristics				
Haemoglobin (g/L)	110.1 ± 15.0	110.2 ± 15.2	110.0 ± 14.4	0.858
Albumin (g/L)	36.8 ± 4.8	36.8 ± 4.6	36.9 ± 5.2	0.706
CRP (mg/L)	4 (2–10)	4 (1–10)	4 (1–10)	0.875
Serum calcium (mmol/L)	2.33 ± 0.17	2.34 ± 0.18	2.31 ± 0.16	0.221
Serum phosphate (mmol/L)	1.56 ± 0.41	1.58 ± 0.41	1.51 ± 0.40	0.057
PTH (ng/L)	27.9 (16.2–42.7)	27 (15.2–42.7)	30.3 (18.6–45.2)	0.731
β2M (mg/L)	23.8 ± 10.9	23.7 ± 9.7	24.1 ± 13.3	0.697
RKF characteristics				
RKF (mL/min/1.73 m^2^)	4.5 (2.4–6.5)	4.5 (2.4–6.4)	4.5 (2.4–7.1)	0.570
Urine output (mL/day)	1156 ± 800	1166 ± 812	1132 ± 771	0.670
Anuria	23 (4.5%)	18 (4.9%)	5 (3.3%)	0.412
Furosemide use	422 (82.5%)	297 (82.2%)	125 (83.3%)	0.773
Dialysis characteristics				
Vintage (months)	2 (2–3)	2 (2–3)	2 (2–3)	0.374
Transport type				**0.038**
Slow	84 (16.4%)	50 (13.8%)	34 (22.6%)	
Average	252 (49.3%)	187 (51.8%)	65 (43.3%)	
Fast	175 (34.2%)	124 (34.3%)	51 (34.0%)	
PD mode				0.955
CAPD	133 (26.1%)	94 (26.1%)	39 (26.3%)	
APD	375 (73.8%)	266 (73.8%)	109 (73.6%)	
Icodextrin use	336 (65.7%)	241 (66.7%)	95 (63.3%)	0.457
Kt/V PD urea	1.12 ± 0.45	1.11 ± 0.44	1.16 ± 0.48	0.262
nPNA (g/kg/day)	0.90 ± 0.24	0.89 ± 0.24	0.92 ± 0.24	0.275
Number of cycles	5.2 ± 2.0	5.1 ± 2.0	5.2 ± 2.0	0.624
Dwell volume (L)	1.80 ± 0.39	1.77 ± 0.33	1.86 ± 0.51	**0.023**

*BMI* body mass index, *SBP* systolic blood pressure, *DBP* diastolic blood pressure, *CRP* C-reactive protein, *PTH* parathyroid hormone, *β2M* β2-microglobulin, *RKF* residual kidney function, *PD* peritoneal dialysis, *CAPD* continuous ambulatory PD, *APD* automated PD

Bold values correspond to *p* < 0.05

### Determination of predictors of β2M

Multivariate analysis included 494 patients without missing values on the considered covariates. In the final multivariate model, factors positively associated with β2M were (Table 2): nPNA, PD mode (APD) and CRP. Factors negatively associated with β2M were: Age, RKF and diabetes. R2 for the final model was 59.7%. Individual contribution to R2 of RKF was 47.7%. Individual contributions to R2 of other variables were between 1.2 and 0.3%. Variables not associated with β2M in the final model were discarded in the following order during backward stepwise procedure: BMI, ethnicity, use of 2.27% dialysate, number of cycles, dwell volume, Davies score, Kt/V PD urea, gender, smoking status, 24-h UF, transport type, use of furosemide, dialysis vintage and use of icodextrin. In a sub-group of patients with 472 patients without missing values on considered covariates, SMMI was added as an independent variable. SMMI was not associated with β2M (*p* = 0.818). Finally, in univariate analysis, nPNA was negatively associated with β2M (*β* = − 28.98, 95% CI = − 42.65 to − 15.30, *p* < 0.001).

**Table 2:** Predictors of β2M

Independent variables	Final model		
	*β*	95% CI	*p* value
Age (years)	− 0.14	− 0.29 to − 0.00	**0.050**
RKF^a^ (mL/min/1.73 m^2^)	− 35.70	− 38.62 to − 32.78	** < 0.001**
PD mode (APD)	9.80	4.58 to 15.02	** < 0.001**
nPNA (g/kg/day)	11.76	1.87 to 21.65	**0.020**
Diabetes	− 6.47	− 11.12 to − 1.81	**0.006**
CRP^b^ (mg/L)	3.31	1.55 to 5.07	** < 0.001**

Multiplied by 100 in order to improve number readability

^a^Square root transformed

^b^Log transformed

*β2M* β2-microglobulin, *RKF* residual kidney function, *CRP* C-reactive protein

### Construction of predictive equation for RKF

During the MFP process, ethnicity and CRP were discarded while other considered predictors were included in the model. Predictive equation for eRKF was given as:

$$\begin{aligned} eRKF\left( {{\text{mL/min/1}}{.73}\;{\text{m}}^{{2}} } \right) = & \frac{45.150}{{\beta 2M^{0.5} }} + \frac{102.419}{{creatinine^{0.5} }} \\ & + 0.037 \times urea - 0.029 \times age \\ & + 0.623 \times gender - 8.733 \\ \end{aligned}$$

where gender is 1 for men and 0 for women. R2 was 56.6% and 55.3% in the modelling and validation groups, respectively.

### Assessment of predictive equation for RKF

**Fig. 1:**
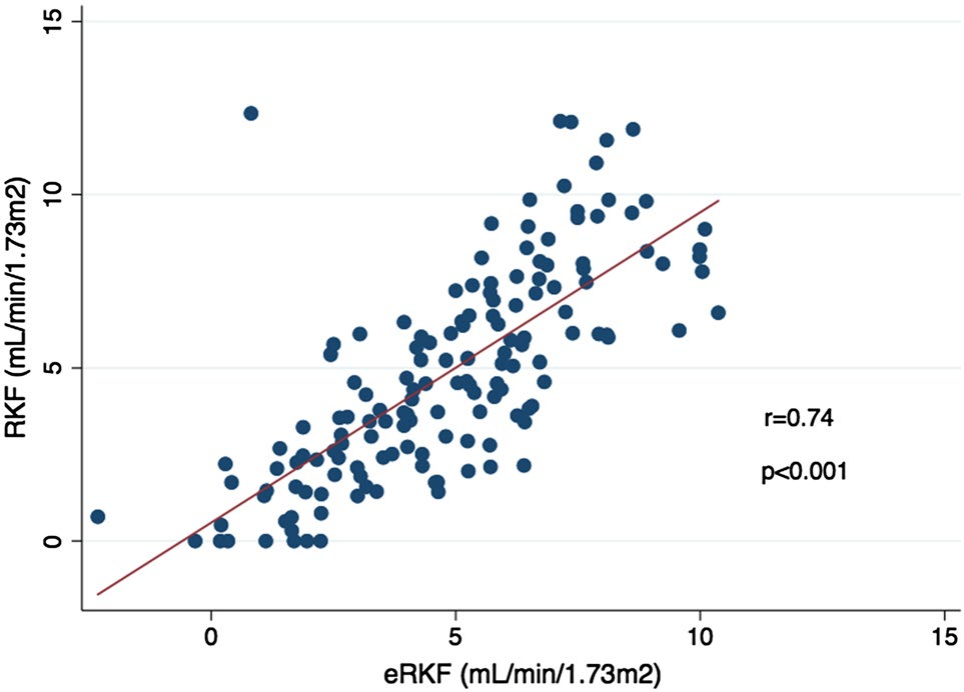
Relationship between RKF and eRKF in the validation group. *RKF* residual kidney function, *eRKF* estimated residual kidney function

**Fig. 2:**
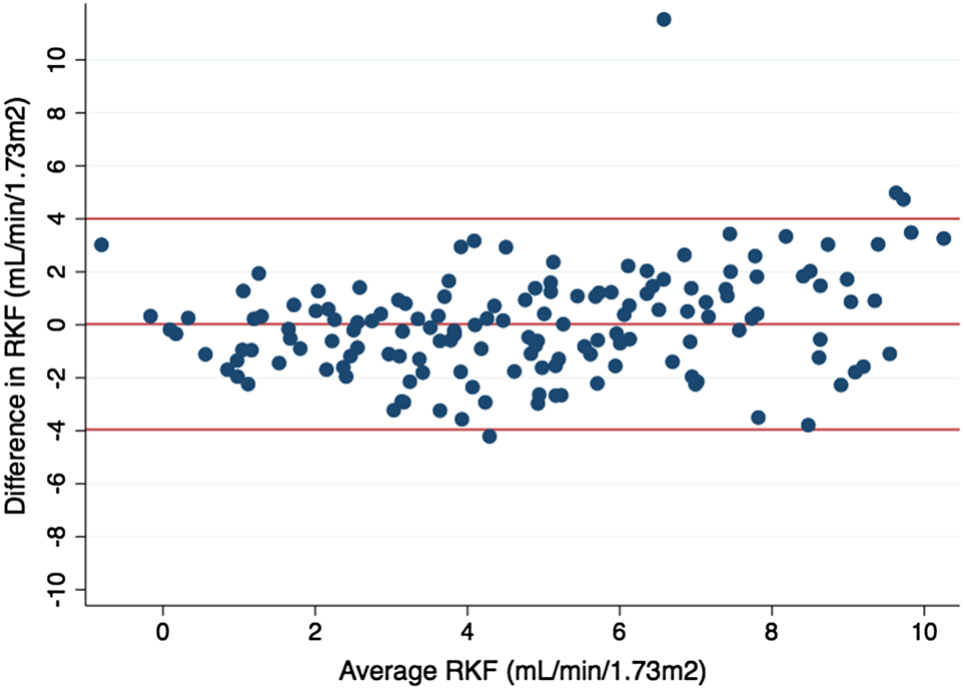
Bland and Altman analysis of RKF and eRKF in the validation group. Difference in RKF is defined as RKF – eRKF. Average RKF is defined as (RKF + eRKF)/2. *RKF* residual kidney function, *eRKF* estimated residual kidney function

**Fig. 3:**
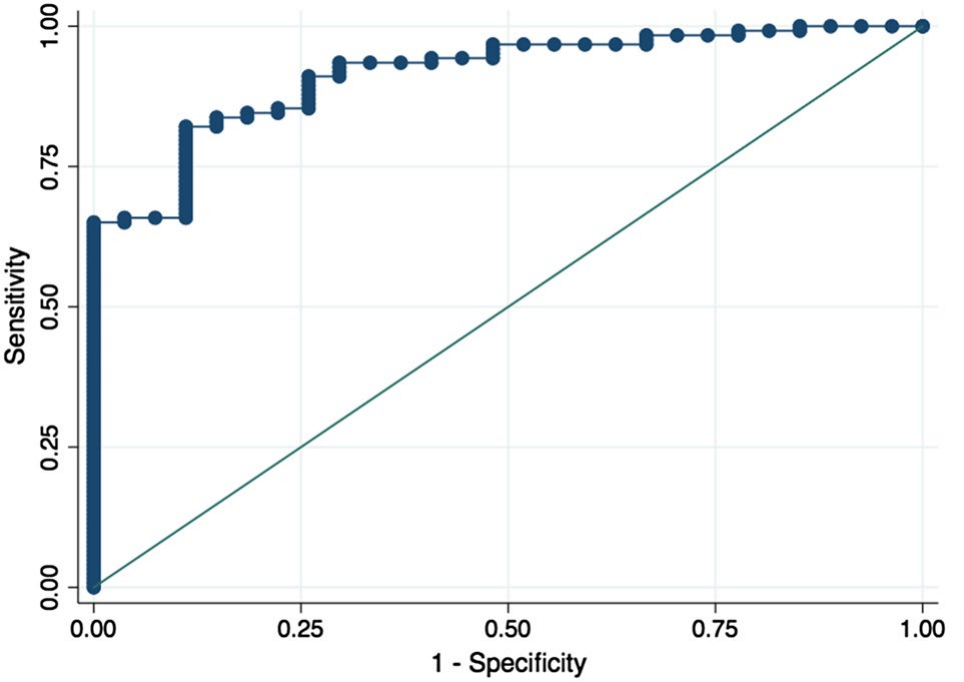
ROC curve for RKF ≥ 2 mL/min/1.73 m^2^ in the validation group. *ROC* receiving operator characteristic, *RKF* residual kidney function

The relationship between eRKF and RKF in the validation group is depicted in Fig. 1. Spearman’s and Pearson’s coefficients were 0.79 and 0.74, respectively (*p* < 0.001 for both). Levels of agreement based on kappa statistics for RKF cut-off values of 1, 2, 3, 4 and 5 mL/min/1.73 m^2^ were 0.44 (moderate), 0.57 (moderate), 0.61 (substantial), 0.61 (substantial) and 0.60 (moderate), respectively. Bland and Altman analysis is depicted in Fig. 2. Bias (95% CI) was − 0.1 (− 0.5 to 0.4) mL/min/1.73 m^2^. Precision (95% CI) and accuracy (95% CI) were 2.5 (2.1 to 2.9) mL/min/1.73 m^2^ and 73 (66 to 80)%, respectively. Area under the curve (AUC) of ROC analysis for RKF cut-off values of 1, 2, 3, 4 and 5 mL/min/1.73 m^2^ were 0.960, 0.915, 0.899, 0.893 and 0.891, respectively. ROC curve for RKF cut-off value of 2 mL/min/1.73 m^2^ is depicted in Fig. 3. Sensitivity and specificity of different eRKF cut-offs to identify different RKF cut-offs are described in Table 3.

**Table 3:** ROC analyses

RKF cut-off to be identified (mL/min/1.73 m^2^)	AUC	95% CI	Predicted eRKF cut-off (mL/min/1.73 m^2^)	Sensitivity (%)	Specificity (%)
1	0.960	0.929 to 0.990	1	97.8	38.5
			2	91.2	84.6
			3	81.0	100
2	0.915	0.865 to 0.964	2	94.3	59.3
			3	85.4	77.8
			4	75.6	88.9
3	0.899	0.849 to 0.949	3	92.2	66.0
			4	82.5	76.6
			5	68.9	89.4
4	0.893	0.842 to 0.943	4	90.5	69.7
			5	78.6	84.8
			6	54.8	92.4
5	0.891	0.839 to 0.942	5	82.9	77.5
			6	64.3	92.5
			7	41.4	100

*ROC* receiving operator characteristic, *RKF* residual kidney function, *AUC* area under the curve, *CI* confidence interval, *eRKF* estimated residual kidney function

## Discussion

In this study, we described predictors of serum β2M levels in patients recently starting PD and developed an equation including small solutes (urea and creatinine) as well as β2M to predict RKF without requiring a 24-h urine collection. While offering moderate precision and accuracy, this equation provided a virtually unbiased estimate of eRKF, and global diagnostic performances based on ROC analysis were higher compared to previous reports [12, 14, 15]. Our results show that β2M could be useful to adapt PD prescriptions in a wide variety of patients and as part of an incremental strategy.

### Predictors of β2M

Serum β2M increases in kidney failure and RKF is more important than peritoneal clearance in determining serum concentration [25]. As such, while peritoneal clearances of small solutes were found to be inversely correlated with their renal clearances, this was not the case for β2M thus suggesting that a loss of RKF could not be simply compensated by an increased peritoneal dialytic clearance of LMWP [16]. Our results are in agreement with these concepts as RKF was the main predictor of serum β2M, with orders of magnitude well above every other significant predictor. The influence of PD prescription on LMWP removal is debated. In a small observational study of 30 patients, CAPD was reported to be more efficient than APD in removing β2M, supporting another observation that β2M peritoneal clearance was greater with a prolonged dwell time, rather than the number of PD exchanges [16, 19]. However, more observational studies, as well as a randomized cross-over study on 15 patients did not demonstrate any difference in peritoneal clearance between CAPD and APD [17, 18]. In our study, while peritoneal β2M clearance was not measured, APD patients had higher β2M concentrations compared to CAPD patients. These results would suggest that dwell time in fact takes precedence over number of exchange in our cohort. In contrast, number of cycles, dwell volume, 24-h UF, use of icodextrin and peritoneal transport type were not predictors of serum β2M levels. Nutritional status was a predictor of β2M in our cohort and patients with higher nPNA values also had higher β2M concentrations. This association is not universally described as β2M was not influenced by nutritional status as assessed by geriatric nutritional risk index score in a sample of 1302 healthy elderly volunteers [26]. However, in line with our findings, another study on 289 HD patients reported a highly significant and positive association between β2M concentration and nPNA [27]. While a definitive mechanistic explanation of such a finding is not allowed by observational studies, hypotheses can be formulated. As β2M levels have previously been positively associated with dialysis vintage, this could indirectly suggest that patients with better nutritional status live longer [28]. Dialysis vintage was however not associated with β2M in our study. Alternatively, as β2M is produced by all cells that express MHC class I genes, it could be a proxy of the overall rate of metabolism and consecutively correlate with nutritional parameters. Finally, it has to be noted that RKF acted as a major confounding factor in the relationship between β2M and nPNA as nPNA was positively associated with β2M in our multivariate model but negatively associated with β2M in univariate analysis. As previously reported, serum β2M was positively associated with CRP, while ethnicity and muscle mass did not have an influence [26].

### Predictive equation for RKF

The main finding of our study is the potential clinical application of a β2M-based equation to predict RKF without having to rely on 24-h urine collections in patients recently starting PD. Compared to previous studies in the field, our equation was built using a different methodological framework. Shafi and colleagues, as well as Steubl and colleagues used conventional spline functions to fit their model [12, 15]. Based on simulation studies, MFP models tend to outperform splines with moderate sample sizes while both methods generally yield similar results on large data sets without local structures [29]. Moreover, MFP models are usually easier to implement owing to their simpler structure and clear criteria for selection of covariates [29].

No single metric can provide an overall assessment of the goodness of fit of a predictive equation and even less of its clinical utility. Our equation could explain 56.6% of RKF variance in the modelling group. Although some variability remains unexplained, overfitting was not a concern as R^2^ was comparable in the validation group. Our equation performed similarly to previous studies on HD patients, with agreement between RKF and eRKF being moderate to substantial depending on the selected cut-off value [30]. Compared to other equations which under- or overestimated RKF, our equation provided a virtually unbiased estimate of RKF since the median difference between measured and estimated values was − 0.1 mL/min/1.73 m^2^ [12, 15]. Moreover, no obvious proportional bias could be detected indicating that the agreement was preserved through the entire range of measurements. However, limits of agreement, as well as precision and accuracy were rather wide in keeping with previous reports using β2M in HD or PD patients although direct comparison with previous equations is not always possible owing to variable definitions of assessment methods and patient selection bias [12, 15, 30]. In our view, such a β2M-based equation would not offer sufficient accuracy to provide a reliable estimate of RKF in PD patients and urine collection would still be necessary when an exact measurement of RKF is required.

Nevertheless, our equation could still be of clinical relevance as part of an incremental strategy in patients initiating PD. ROC analysis globally demonstrated high diagnostic capabilities, and we could detect a RKF ≥ 2 mL/min/1.73 m^2^ with a 0.915 AUC, an improvement compared to previous reports for a similar cut-off [12, 14, 15]. Detection of other RKF cut-offs could also prove of clinical interest. As an example, a predicted eRKF > 1 mL/min/1.73 m^2^ had a 97.8% sensitivity to detect a RKF > 1 mL/min/1.73 m^2^, thus giving reasonably high certainty that the true RKF is below 1 mL/min/1.73 m^2^ when the predicted eRKF is below this threshold, so that such patients could be reliably excluded from an incremental strategy without urine collections. At the opposite end of the spectrum, predicted eRKF > 7 mL/min/1.73 m^2^ had a 100% specificity to detect a RKF > 5 mL/min/1.73 m^2^ and serum β2M could formally replace urine collection to identify such patients. As current clinical guidelines recommend quarterly RKF measurement for PD patients, serum β2M levels could potentially be used during follow-up of an incremental prescription to ascertain that RKF remains above a certain pre-defined value. This would allow serial monitoring of RKF in patients who are unable to collect their urine due to problems with incontinence or other disabilities. Potentially, such an approach could also help reduce the impact of the important inherent variability of RKF measurement based on urine collection in clinical practice [31].

Compared to previous reports, our study differs in several aspects. First, our cohort consisted of a heterogeneous population of outpatients recently starting PD presenting with a wider range of RKF values [12, 15, 30]. This could potentially introduce a conservative bias in the diagnostic performances of our model. Second, as PD modality is chosen according to individual characteristics at out-centre, both CAPD and APD patients were well represented. Despite potential differences in middle molecule clearances between modalities, our equation applies equally to CAPD as well as APD patients. Finally, since precise and clinically useful estimates of RKF based on equations using serum molecules is probably unrealistic at the present time, we focused on a threshold-based strategy potentially applicable to incremental PD.

### Limitations

All studies have limitations. First, the mean of creatinine and urea clearances based on 24-h urine samples was used as the reference test for RKF measurement. As this reflects only small solutes clearance, it may differ from RKF measured with exogenous markers. Moreover, urine collection was not supervised and errors cannot be excluded. However, the purpose of this study was to assess the performance of a serum-based equation compared to the routinely used RKF measurement based on a 24-h urine collection. Second, serum β2M levels have been reported to increase as an acute phase reactant in auto-immune inflammatory as well as lymphoproliferative diseases [26]. Although, similarly to previous reports, we excluded patients with active malignancies, we did include patients with a range of CRP values that were adjusted for in our analyses [15]. Moreover, other studies have not reported an association between malignancy and β2M levels in dialysis patients [30]. Finally, as longitudinal data were not available, the performances of our equations to detect changes in RKF could not be assessed. While shared with similar studies on the subject, this limitation hampers definitive conclusions on the clinical relevance of our approach as part of a real-life incremental strategy.

## Conclusion

In conclusion, we developed an equation to predict RKF without requiring a 24-h urine collection based on serum β2M, a relatively widely available biomarker, in patients recently initiating PD treatment. While this approach might not be accurate enough to entirely replace standard measurement of RKF using 24-h urine collection, it could prove useful in guiding prescription based on selected eRKF cut-off values as part of an incremental dialysis strategy, as well as for serially monitoring RKF in patients unable to reliably collect their urine. Further studies are required to determine whether PD prescription could be safely guided by such equations.

## Data Availability

Available from corresponding author upon reasonable request.
